# Special Issue: “Gut Microbiota and Nutrition in Human Health”

**DOI:** 10.3390/ijms252111589

**Published:** 2024-10-29

**Authors:** Sunmin Park

**Affiliations:** 1 Department of Bioconvergence, Hoseo University, Asan 31499, Republic of Korea; smpark@hoseo.edu; 2 Department of Food and Nutrition, Obesity/Diabetes Research Center, Hoseo University, Asan 31499, Republic of Korea

## 1. Background

The microbiome is a singular term for a vast array of life forms that live symbiotically within the bodies of human and animal hosts, forming commensal, parasitic, or mutualistic relationships [1]. Human hosts have consumed foods that include bacteria and other microorganisms that can become part of their microbiome for a long time. They also consume foods that may contribute nutrients that support their microbiome [1]. Such foods include fermented foods with live bacteria and yeasts and plant foods composed of polysaccharides that are only digestible by gut microbiota. In recent years, people have deliberately included live microorganisms (probiotics) and foods to increase the proliferation of specific microbes (prebiotics).

It is not surprising that the microbiome profoundly affects many, if not most, of the human body’s systems. The colonic microbiome is known to contain more cells than the human body and over 150 times as many genes as the entire human genome [2,3]. Although much of the microbiome remains fairly constant, it is also known that the microbiome is not a static system, but rather a fluid collection of life forms that can undergo changes as it responds to different conditions within its environment [4] and maybe a biomarker of disease or an agent for change [5,6].

**Figure 1:**
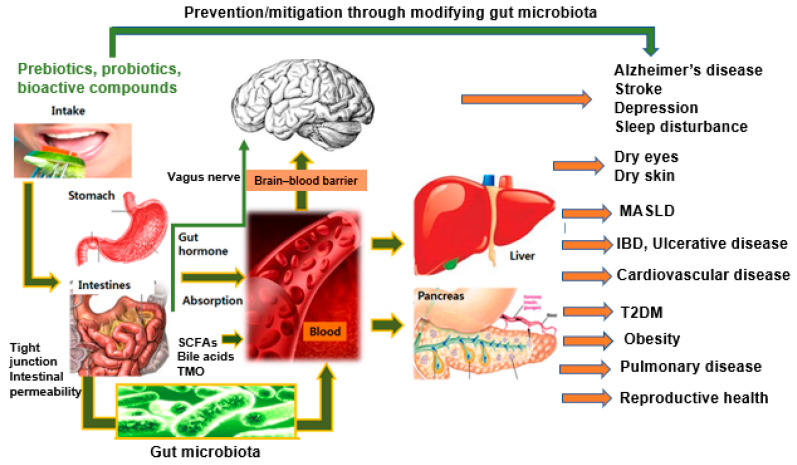
The gut–brain–body axis: prevention and mitigation of diseases through gut microbiota modulation. This diagram illustrates the complex interactions between gut microbiota and various body systems, highlighting how modifying the gut microbiome using prebiotics, probiotics, and bioactive compounds can potentially prevent or mitigate a wide range of diseases. The image shows the pathway from the intake of these compounds through the digestive system, their effects on gut microbiota, and the subsequent impacts on different organs and health conditions. SCFAs, short-chain fatty acids; TMO, trimethylamine N-oxide; MASLD, metabolic dysfunction-association steatotic liver disease; IBD, irritable bowel disease; T2DM, type 2 diabetes mellitus.

Of particular interest is the gut–brain axis, a bidirectional communication system between the gastrointestinal tract and the central nervous system [7]. This axis involves complex interactions among the enteric nervous system, the autonomic nervous system (especially the vagus nervous system), and the neuroendocrine system [7,8]. The gut microbiome plays a crucial role in this axis, influencing brain function and behavior through various mechanisms, including the production of neurotransmitters, modulation of the immune system, and regulation of the hypothalamic–pituitary–adrenal (HPA) axis [9,10]. The gut–brain axis is connected to other organ activities, such as the gut–brain–liver axis, and dietary components can modulate the axis by changing the composition and interactions of gut microbiota (Figure 1) [11,12,13].

Despite recent advances in discovering the physiological effects of the microbiome on the host, a rather limited understanding of the functional consequences of microbiome variations, especially the impact of specific bacterial species within the microbiome, remains. This Special Issue of the *International Journal of Molecular Sciences* seeks to clarify the physiological, pathological, and even pharmacological implications of the human gut microbiome. This Special Issue also clearly illustrates the great variability in gut microbes, their plethora of effects, and, thus, the multifarious microbiome. By exploring these diverse aspects, we aim to stimulate further research and collaboration in this rapidly evolving field, ultimately contributing to developing novel therapeutic strategies and personalized interventions for improving human health through microbiome modulation.

## 2. Key Themes and Insights

### 2.1. Diet as a Modulator of Gut Microbiota

Through the studies included in this Special Issue, one overarching message becomes clear [14,15]: the nutrients in an individual’s diet act as a powerful modulator of the gut microbiota, which in turn affects systemic health outcomes; however, the host’s genetics, including the capabilities of their autonomous nervous system, bile acid reabsorption, and neurotransmitters, influence the impact of nutrients on the gut microbiota [14]. As the gut microbiome emerges as a key player in conditions ranging from metabolic disorders to cognitive health, understanding how specific dietary components influence this complex microbial environment has never been more critical [15].

### 2.2. Beyond the Gut: Systemic Effects of Microbiota–Nutrition Interactions

One key insight from the papers is that the influence of the gut microbiota extends beyond digestive health. The microbiome impacts various systems, including immune responses, neurological functions, and reproductive health [16]. The research in this Special Issue demonstrates that modulating gut bacteria can offer unexpected benefits, such as improving dry eye symptoms, enhancing sleep quality, or promoting reproductive health by optimizing the gut flora [10,17,18,19,20]. This growing body of evidence highlights the importance of understanding the broad systemic effects of microbiome–nutrition interactions.

### 2.3. Linking Food Components with Microbiota Metabolism

The studies in this Special Issue also emphasize how specific nutritional components interact with gut microbes [21,22]. The metabolic capacity of the gut microbiota to process gluten opens new avenues for understanding how everyday dietary elements influence microbial diversity and health [21]. This insight paves the way for more personalized dietary recommendations, tailored to individual microbiome profiles, which could lead to more effective prevention and treatment strategies.

### 2.4. Therapeutic Potential of Microbiota Modulation

A recurring theme is the therapeutic potential of using diet to modulate the gut microbiota. The papers in this Special Issue suggest that nutritional interventions could offer viable strategies for improving metabolic conditions, enhancing immune responses, and addressing various health challenges [10,18,23,24]. By harnessing the microbiota’s therapeutic potential, these studies present compelling evidence that the dietary component, as a modifiable factor, holds promise for addressing microbiome-related disorders [11,12,25].

## 3. Overview of Featured Studies

This Special Issue showcases a diverse range of studies that significantly advance our understanding of the gut microbiome’s role in human health. In the realm of methodological advances, Carnicero-Mayo et al. [21] introduced a novel approach for culturing and maintaining stable gut microbial communities derived from gluten metabolism. This innovative method provides researchers with a valuable tool for studying the intricate dynamics of the gut microbiota with regard to gluten consumption, opening up new possibilities for understanding how dietary components interact with gut microbes and influence host health.

Several studies in this Special Issue focused on disease-specific investigations, describing the role of gut microbiota in various health conditions [7,20,24]. Cisek et al. [24] delved into the often-overlooked archaeal component of the gut microbiome in pediatric inflammatory bowel disease (IBD). Their findings revealed significant decreases in total methanogens in both Crohn’s disease and ulcerative colitis compared with healthy controls, highlighting the potential role of methanogenic archaea in IBD’s pathogenesis and progression. In another study, Sato et al. [20] employed non-invasive methods to evaluate the relationship between liver fibrosis and gut microbiota in the general population. Their research uncovered significant differences in microbial diversity between individuals with and without liver fibrosis, suggesting a potential link between the gut microbial composition and liver health. This finding could have important implications for both the diagnosis and treatment of liver diseases. Exploring the gut–brain axis, Park et al. [7] investigated gut microbiota alterations in depression. By classifying subjects based on enterotypes, they revealed significant differences in the microbial composition and associated metabolic pathways between depressed individuals and healthy controls, contributing to our understanding of the complex relationship between gut microbiota and mental health.

This Special Issue also featured several intervention studies, demonstrating the potential of microbiome-targeted therapies across diverse health conditions. Wiacek et al. [10] conducted a double-blind clinical trial in dancers, investigating the effects of *L. helveticus* and *B. longum* supplementation on the endocannabinoid system and sleep quality. While the intervention did not significantly impact endocannabinoid markers, it did improve sleep quality, suggesting potential cognitive benefits of probiotics that may extend beyond their direct effects on the gut microbiota. In an innovative study exploring the gut–eye axis, Lee et al. [17] orally supplemented *Limosilactobacillus fermentum* to mice with corneal injury. The probiotic not only altered the gut microbiota and decreased gut inflammation but also reversed the “dry eye” symptoms and corneal inflammation associated with the eye injury model. This improvement appeared to be mediated by the modulation of matrix metalloproteinase, highlighting the far-reaching effects of gut microbiome interventions. Expanding the scope of microbiome research to reproductive health, Huang et al. [18] administered strontium chloride to young male rats. This intervention led to a decreased abundance of gut bacteria that are known to adversely affect reproductive health, while simultaneously increasing the sperm quality and testosterone levels in the mice. These findings open up new avenues for exploring the gut microbiome’s role in reproductive health and fertility.

## 4. Future Directions in Gut Microbiota and Nutrition Research

As research on the gut microbiota and its impact on human health advances, several key areas emerge as crucial for further exploration. A deeper understanding of the signaling pathways that mediate the interactions between diet, the gut microbiome, and host health is essential and linked to the topic of enterotypes [26]. Identifying these pathways will clarify how specific dietary components and bioactive compounds influence the composition and function of the microbiome, potentially leading to targeted nutritional interventions for improving health outcomes. In addition, there is a growing need to explore the gut–organ axis, particularly the gut–brain axis, and its broader implications for various diseases [27,28]. Increasing evidence points to the microbiome’s role in modulating not only gastrointestinal, metabolic, and immune functions but also neurological processes, with potential implications for conditions such as neurodegenerative disorders, mental health conditions, diabetes, and cardiovascular diseases in different ethnicities [26,29,30]. Future studies should focus on elucidating these complex interactions to uncover new therapeutic opportunities.

Furthermore, the variability in individual microbiomes underscores the potential of personalized nutrition strategies. Tailoring dietary interventions based on an individual’s unique microbiome profile could pave the way for more effective approaches to preventing and managing conditions such as obesity, diabetes, and gastrointestinal disorders. This precision nutrition approach promises to transform healthcare by leveraging microbiome data for personalized dietary recommendations. Together, these future research directions will help unlock the full potential of nutritional interventions that modulate the gut microbiome, leading to more precise and effective strategies for enhancing human health. The insights gained from the studies in this Special Issue provide a solid foundation for continued exploration in these areas.
